# MSWF: A Multi-Modal Remote Sensing Image Matching Method Based on a Side Window Filter with Global Position, Orientation, and Scale Guidance

**DOI:** 10.3390/s25144472

**Published:** 2025-07-18

**Authors:** Jiaqing Ye, Guorong Yu, Haizhou Bao

**Affiliations:** School of Computer Science and Technology, Wuhan University of Science and Technology, Wuhan 430081, China; yjq@wust.edu.cn (J.Y.); baohaizhou@wust.edu.cn (H.B.)

**Keywords:** multi-modal remote sensing images, feature matching, feature descriptor, side window filter

## Abstract

Multi-modal remote sensing image (MRSI) matching suffers from severe nonlinear radiometric distortions and geometric deformations, and conventional feature-based techniques are generally ineffective. This study proposes a novel and robust MRSI matching method using the side window filter (MSWF). First, a novel side window scale space is constructed based on the side window filter (SWF), which can preserve shared image contours and facilitate the extraction of feature points within this newly defined scale space. Second, noise thresholds in phase congruency (PC) computation are adaptively refined with the Weibull distribution; weighted phase features are then exploited to determine the principal orientation of each point, from which a maximum index map (MIM) descriptor is constructed. Third, coarse position, orientation, and scale information obtained through global matching are employed to estimate image-pair geometry, after which descriptors are recalculated for precise correspondence search. MSWF is benchmarked against eight state-of-the-art multi-modal methods—six hand-crafted (PSO-SIFT, LGHD, RIFT, RIFT2, HAPCG, COFSM) and two learning-based (CMM-Net, RedFeat) methods—on three public datasets. Experiments demonstrate that MSWF consistently achieves the highest number of correct matches (NCM) and the highest rate of correct matches (RCM) while delivering the lowest root mean square error (RMSE), confirming its superiority for challenging MRSI registration tasks.

## 1. Introduction

With the rapid development of modern information technology, sensors across various platforms, such as optical [1], infrared [2], synthetic aperture radar (SAR) [3], and light detection and ranging (LiDAR) [4], continue to emerge, providing diverse modalities of data sources for Earth observations. Compared to single-sensor observation data, MRSI offers redundant, complementary, and synergistic information. Effectively integrating multi-sensor and multi-modal remote sensing data is a challenge in the field of photogrammetry and remote sensing. Specifically, multi-modal image matching enables the integration of images obtained from different sensors into the same coordinate system, allowing for the extraction of high-quality information suitable for various applications. Through the joint processing of multi-modal data, more accurate, complete, and reliable observation results can be achieved compared to single-sensor data. This integration is a fundamental prerequisite for numerous collaborative processes and integrated applications in photogrammetry and remote sensing. Therefore, a robust MRSI matching [5,6,7,8] method is crucial for high-precision aerial triangulation, 3D terrain reconstruction, and multi-modal image fusion.

To address the challenges of MRSI matching, a range of novel methodologies has been proposed. These approaches can be roughly classified into three categories: region-based matching methods, feature-based matching methods, and deep learning-based methods [9,10]. Region-based registration methods typically select an appropriate similarity metric together with a specific optimization method to estimate geometric transformation parameters. Their performance is largely determined by the choice of similarity metrics, such as the sum of squared differences (SSD) [11], the normalized cross correlation (NCC) [12], and mutual information (MI) [13]. These metrics have been widely applied in image registration but are not suitable for MRSI matching due to the significant NRDs. To address these limitations, some studies have focused on similarity computation based on structural features rather than direct image intensities, employing methods such as local self-similarity (LSS) [14], dense local self-similarity (DLSS) [15], histogram of orientated PC (HOPC) [16], and rank-based local self-similarity (RLSS) [17]. Additionally, pixel-by-pixel channel features of orientated gradients (CFOGs), combined with the fast Fourier transform [18], have been introduced to accelerate computation and effectively mitigate NRDs. Despite these advancements, region-based methods require prior attitude parameters and are sensitive to geometric distortions.

Unlike region-based methods, feature-based methods process extracted features instead of the whole image, which can largely decrease the computational requirements. Among these methods, SIFT [19] is the most widely applied method; however, it often fails to match MRSI due to significant modality differences. This has led to the development of various improved algorithms. Dellinger et al. proposed SAR-SIFT [20], which modifies the gradient extraction process of SIFT by incorporating the exponentially weighted average (ROEWA) ratio. Xiang et al. proposed OS-SIFT [21], which enhances SIFT by employing a scale-invariant feature extraction method. Ma et al. developed a SIFT-based descriptor that effectively captures features within the SIFT framework. Additionally, Ma et al. [22] incorporated the particle swarm optimization (PSO) algorithm into SIFT, optimizing the key point matching process and enhancing the accuracy and robustness of feature matching. These SIFT-based methods are effective for multi-spectral or single-modal images with linear distortion differences. Additionally, the preservation of coexisting contours in multi-modal images remains a prominent research focus. Yao et al. [23] employed co-occurrence filtering to construct a scale space and combined the Butterworth filter with the Sobel operator to refine the image gradient, further enhancing the robustness of multi-modal image descriptions and mitigating the influence of NRDs. Nevertheless, the feature maps in these methods still primarily rely on gradient information, which lacks robustness to substantial nonlinear distortions, complex radiometric changes, and noise. To address these limitations, many researchers have developed feature descriptors based on PC [24,25] to mitigate the effects of NRDs. For instance, Ye et al. proposed the (minimum PC moment)-Lap (MMPC) feature detector, utilizing a PC model with illumination and contrast invariance, along with the local directional PC histogram (LHOPC) feature descriptor, which leverages both the magnitude and orientation of PC. This approach, inspired by the spatial configuration of DAISY, calculates local invariant features [5]. Additionally, Li et al. proposed the radiation variation insensitive feature transform (RIFT) [26], which uses Log-Gabor convolution sequences to construct MIM descriptors, thereby improving feature detection stability and enhancing the radiometric invariance of feature descriptions. Yao et al. proposed a method that combines anisotropic weighted moments with the histogram of absolute PC gradients (HAPCG) [27], significantly improving the robustness of image matching. Zhang et al. proposed a matching technique based on the weighted phase orientation histogram (HOWP), which replaces traditional image gradient orientation features with a weighted phase orientation model that utilizes principal component energy components with the largest and smallest bandwidths, thereby further enhancing the robustness of MRSI matching [28]. Hou et al. developed a novel multi-modal image matching algorithm, POS-GIFT [29], which utilizes a multi-layer circular point sampling pattern to effectively capture local image structures and integrates multi-directional, multi-scale Log-Gabor filter results to propose a robust feature descriptor capable of handling rotational variations and intensity distortions. This method enables high-confidence matching even under complex nonlinear intensity, scale, and rotational transformations. Although these PC-based methods have substantially improved MRSI registration, the accuracy of registration remains somewhat limited. This limitation arises from the fact that PC generates artifacts at feature boundaries and is particularly sensitive to noise, which hinders the reliable extraction of effective features and negatively impacts both localization accuracy and feature uniqueness. Consequently, the development of highly repeatable and distinctive features remains a critical challenge for feature-based registration methods.

In recent years, deep learning technology has developed rapidly and has been increasingly used in MRSI matching [30]. Ye et al. [31] studied features from fully connected and convolutional layers with varying aggregation sizes in convolutional neural networks (CNNs), combining these deep features with SIFT for successful multi-sensor remote sensing image registration. Ma et al. [32] proposed a coarse-to-fine two-stage registration framework that combines CNNs and local feature technology. Feature matching and transformation estimation are refined by combining CNN-derived features and hand-crafted local features. Hughes et al. [33] proposed a three-stage matching framework composed of a goodness network, a multi-scale matching network, and an outlier reduction network, enabling fully automated multi-scale SAR and optical image registration. Lan et al. proposed a cross modality matching net (CMM-Net), which detects and describes feature points on high-dimensional feature maps of multi-modal remote sensing images extracted by CNNs. Wang et al. proposed Matchformer [34], which incorporates a lightweight decoder utilizing multi-scale features to reduce computational cost and employs a cross-attention mechanism to enhance robustness and matching accuracy. Xie et al. [35] designed a semantically guided (SemLA) network for cascaded matching, alignment, and fusion of infrared and visible light imagery. Yang et al. [36] proposed a lightweight pseudo-Siamese convolutional neural network with multi-scale fusion and noise resistance, achieving higher accuracy, robustness, and real-time speed than existing matching methods. Zhu et al. [37] proposed a viewpoint-invariant deformable feature transformation with deformable convolutions and seed attention, achieving superior robustness and accuracy in aerial and ground image matching. Lin et al. [38] proposed a heterogeneous model fitting technique combining multi-orientation phase consistency, log-polar descriptors of variable sizes, and integrated transformations, achieving superior multi-source image correspondence accuracy across seven datasets. Yang et al. [39] introduced an adjacent self-similarity three-dimensional convolution strategy with block-based corner detection to improve the robustness and efficiency of multi-modal image registration. Ye et al. [40] proposed attention-enhanced structural features with multi-branch global attention and multi-cropping matching loss, boosting optical–synthetic aperture radar registration accuracy by up to 8.7 percent. Despite the significant advancements achieved by deep learning-based matching methods, these approaches generally require extensive labeled training datasets and substantial hyperparameter tuning, both of which are time-consuming and computationally demanding. Moreover, their generalization capabilities and practical applicability remain limited by the inherent complexity and variability of remote sensing data.

The side window filter (SWF) [41] is proposed to effectively preserve edges and maintain image contours. Traditional filters such as Gaussian filters and mean filters place the filter window at the center of the pixel to be processed, resulting in the influence of neighboring pixels on both sides of the edge when filtering edge pixels. This causes the edge information to diffuse along the edge of normal orientation, making the image contour blurred, which even the anisotropic diffusion filter [42] cannot completely alleviate. To address this limitation, SWF positions the pixel to be processed at the edge of the filter window, ensuring that the window is predominantly aligned on one side of the edge and preventing the filter window from crossing the edge. Additionally, SWF is characterized by a minimal number of parameters, ease of implementation, and robust edge-preserving performance for images of different modalities. When applied to MRSI matching, SWF effectively mitigates NRDs and improves edge feature extraction. Consequently, the image scale space constructed using SWF preserves image contours more effectively, thus enhancing multi-modal image matching performance.

This study proposes a feature-based MRSI matching method, and the main contributions are as follows:

(1) The proposed side window scale space using SWF preserves contour information and increases the number of feature points extracted from most modalities.
(2) To address the sensitivity of the PC to noise, a new noise threshold estimation method is proposed using the Weibull distribution to better estimate noise levels and reduce the impact of noise on MRSI matching.
(3) A novel global-to-local cascade matching strategy based on the newly constructed image scale space is proposed. Guided by the results of global matching, the consistent position, orientation, and scale features of corresponding relationships are used to locally regenerate feature descriptors, performing cascade matching. Experimental results show that this significantly improves the accuracy and reliability of the matching results.

## 2. Methodology

**Figure 1:**
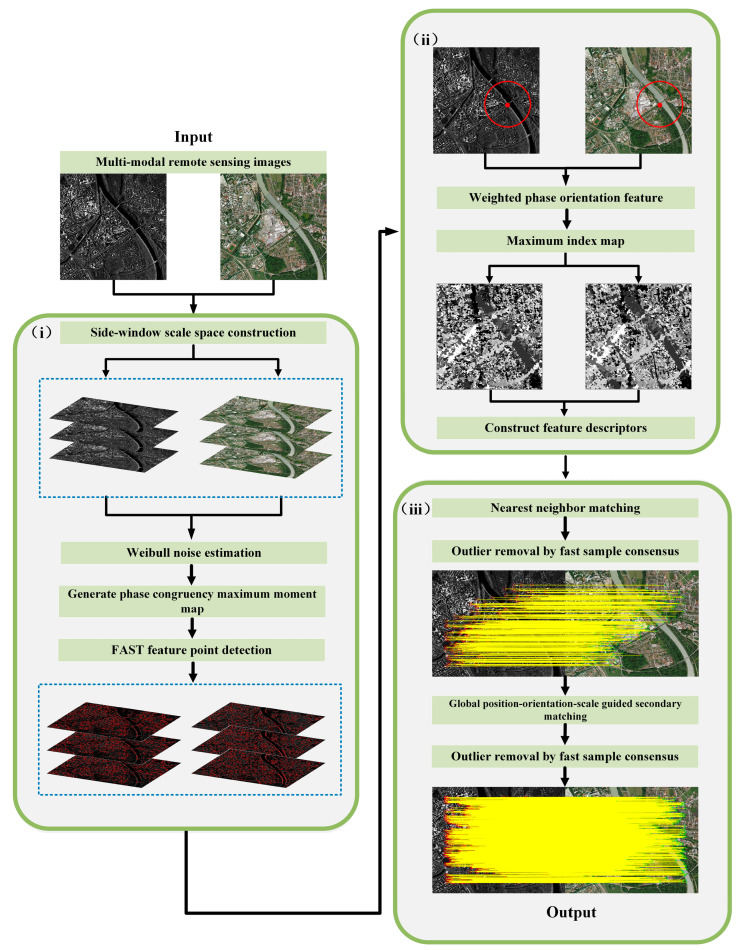
(**i**–**iii**) The flowchart of the MSWF.

This section details the proposed MSWF method, focusing on feature detection and description. First, a novel side window scale space is constructed to better address scale differences and leverage edge information for feature point extraction. Next, a novel noise estimation method is introduced to enhance the robustness of PC against noise. Lastly, a cascade matching strategy, guided by global position, orientation, and scale, is proposed based on initial global matching. The flowchart of the algorithm is shown in Figure 1.

### 2.1. Side Window Scale Space Construction

Scale spaces can be constructed using two main approaches: linear and nonlinear. For example, the classic SIFT [19] constructs a scale space by convolving an image with Gaussian kernels of different sizes and then downsampling in a pyramidal manner. However, the Gaussian filter cannot differentiate between uniform and edge regions when applying the same scale filter to both, resulting in a significant loss of edge information. To address this issue, the theory of nonlinear scale spaces was introduced. Traditional nonlinear scale spaces typically use isotropic [43] or anisotropic diffusion filtering. Although nonlinear scale spaces preserve more edge information compared to linear scale spaces, edge information loss cannot be avoided. In particular, the NRDs of MSRI are more obvious than that of other modalities. Therefore, it is crucial to preserve edge features and develop a scale space that effectively mitigates nonlinear distortion.

**Figure 2:**
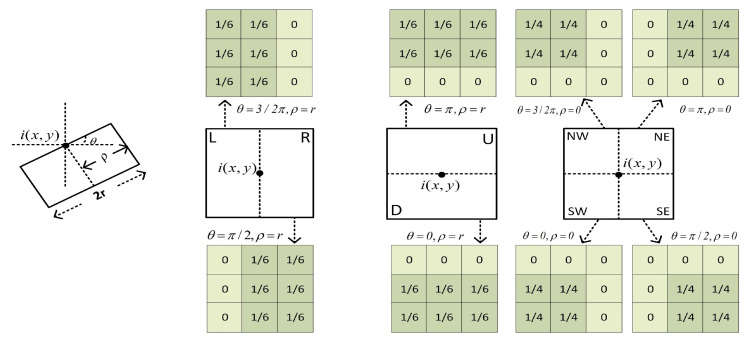
Definition of the side window. Here, *r* is the radius of the window.

The definition of the side window is shown in Figure 2, with parameters θ and r. Here, θ represents the angle between the window and the horizontal line, r represents the window radius, and ρ∈{0,r} and (x,y) represent the position of the target pixel i. In addition, r is a user-defined parameter, which is set to 6 in this paper. By changing θ and fixing (x,y), the orientation of the window is altered while aligning its side with the orientation.

In order to reduce the computational complexity, eight side windows are defined, as shown in Figure 2. The orientations of these eight windows correspond to the θ=d×π/2,d∈[0,3] with two different values for ρ. By setting ρ=r, the down ($\omega_{i}^{D}$), right ($\omega_{i}^{R}$), up ($\omega_{i}^{U}$), and left ($\omega_{i}^{L}$) side windows are obtained. They align i with the sides. By setting ρ=0, the southwest ($\omega_{i}^{SW}$), southeast ($\omega_{i}^{SE}$), northeast ($\omega_{i}^{NE}$), and northwest ($\omega_{i}^{NW}$) side windows are obtained, as shown in Figure 2. They align i with their respective corners.

Eight outputs are obtained by applying the filter kernel F in each side window. This is represented as $I_{i}^{\prime \theta ,\rho }$, where θ=d×π/2, d∈[0,3] and ρ∈{0,r}.

(1) $$I_{i}^{\prime \theta ,\rho ,r}=F(q_{i},\theta ,\rho ,r)$$

To better preserve the edge, the difference before and after filtering at the edge should be minimized. Specifically, the side window filter output is highly similar to the input at the edge. Therefore, by enumerating multiple directions, the side window output with the smallest $L_{2}$ distance from the input intensity is finally selected as the final output.

(2) $$I_{SWF}^{\prime }=arg\underset{\theta ,\forall I_{i}^{\prime \theta ,\rho ,r}}{\min}||q_{i}-I_{i}^{\prime \theta ,\rho ,r}|{|}_{2}^{2}$$

In Equation (2), $I_{SWF}^{\prime }$ is the output of the SWF. Equation (2) is referred to as the SWF technique.

The side window scale space of the current image layer is obtained using Equations (1) and (2). The traditional scale space usually constructs an image pyramid by downsampling. In such scale spaces, the size of each layer of the image decreases rapidly during the downsampling process, resulting in the loss of image texture information. Based on this notion, the scale space in this paper does not downsample the image. This not only retains the texture information, but also reduces the construction difficulty and computational complexity of the scale space. The scale space is divided into layers (generally no more than 6 layers), and the filter kernel definition and pixel values for each layer are shown in the equations below.

(3) $$\left\{\begin{array}{l} k_{n}=\frac{1}{{\left(2k_{0}+1\right)}^{2(n-1)}},(n=1,2\ldots S)\\ f_{n}(x,y)=k_{n}\sum_{(s,t)\in S_{xy}}g(s,t),n\geq 1 \end{array}\right.$$

**Figure 3:**
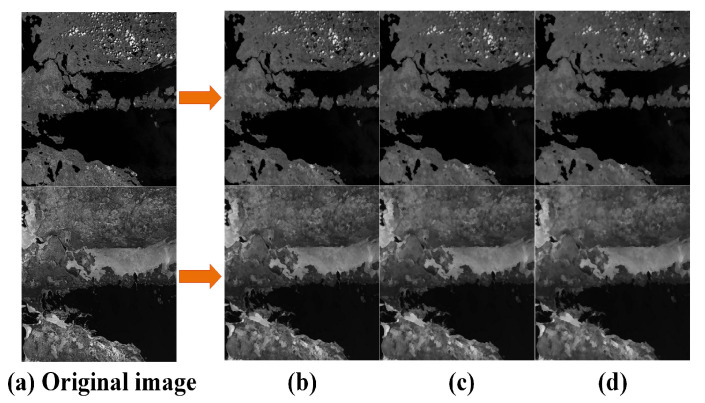
MRSI of side window filtering (SWF): (**a**) is the original image; (**b**) is the second layer result of the SWF scale space; (**c**) is the third layer result of the SWF scale space; and (**d**) is the fourth layer result of the SWF scale space.

In Equation (3), $k_{n}$ represents the filter kernel of the n−th layer of the scale space; $k_{0}$ is the radius of the filter kernel, with an empirical value of 6; S represents the number of scale space layers in the multi-modal image; $f_{n}(x,y)$ is the pixel value of the target pixel i(x,y) at the n−th layer of the image; and $S_{xy}$ represents all the pixels within the filter window centered at (x,y); g(s,t) represents the initial image. The constructed scale space is shown in Figure 3.

### 2.2. Feature Extraction

Traditional feature matching methods typically rely on image intensity or gradient information in the spatial domain, which are not sufficiently robust to handle NRDs and geometric variations. On the other hand, frequency domain information, such as phase information, can represent multi-modal images more effectively. This approach assumes that features can be perceived at the maximum location of Fourier components. Since PC is independent of signal amplitude, it offers greater robustness to radiometric differences than gradient-based methods. Morrone and Owens [44] demonstrated that points with highly consistent local phase information elicit strong responses in the human visual system. The degree of consistency of local phase information at different angles is referred to as the PC measure.

#### 2.2.1. Phase-Congruency Computation

Given that image data are a two-dimensional signal, Kovesi proposed performing one-dimensional analysis in multiple orientations and combining the results to obtain PC. A two-dimensional filter can be derived by applying Gaussian propagation to the vertical orientation of the one-dimensional Log-Gabor filter. Specifically, the tangential component is constructed using the original one-dimensional Log-Gabor filter, while the radial component is generated with a Gaussian transfer function.

(4) $$\left\{\begin{array}{l} G(\theta )=\exp(\frac{-{\left(\theta -\theta_{0}\right)}^{2}}{2\sigma_{\theta }^{2}}\\ LG(\hat{w})=\exp(\frac{-{\left(\ln(\hat{w}/{\hat{w}}_{0})\right)}^{2}}{2{\left(\ln(\sigma_{\hat{w}}/{\hat{w}}_{0})\right)}^{2}}) \end{array}\right.$$

In Equation (4), $\theta_{0}$ is the filtering orientation, $\sigma_{\theta }$ is the standard deviation of the Gaussian function, ${\hat{w}}_{0}$ is the center frequency of the Log-Gabor filter, and $\sigma_{\hat{w}}$ is the coefficient related to the bandwidth. Therefore, the formula for the two-dimensional frequency domain Log-Gabor filter is expressed as follows:

(5) $$LG(\hat{w},\theta )=LG(\hat{w})G(\theta )$$

Given I(x,y) as the two-dimensional image signal, $M_{n\bar{o}}^{e}$ and $M_{n\bar{o}}^{o}$ denote the even-symmetric and odd-symmetric Log-Gabor wavelets, respectively, at the n−th scale and $\bar{o}-th$ orientation. By convolving these two wavelet functions with the image signal, the response components $e_{n\bar{o}}(x,y)$ and $o_{n\bar{o}}(x,y)$ are obtained:

(6) $$\left[e_{n\bar{o}}(x,y),o_{n\bar{o}}(x,y)]=[I(x,y)*M_{n\bar{o}}^{e},I(x,y)*M_{n\bar{o}}^{o}\right]$$

The amplitude and phase of the image after the wavelet transform at scale n and orientation $\bar{o}$, respectively, are given as follows:

(7) $$A_{n\bar{o}}(x,y)=\sqrt{e_{n\bar{o}}{\left(x,y\right)}^{2}+o_{n\bar{o}}{\left(x,y\right)}^{2}}$$

(8) $$\phi_{n\bar{o}}(x,y)=\arctan(o_{n\bar{o}}(x,y)/e_{n\bar{o}}(x,y))$$

Taking the orientation information into consideration, the improved two-dimensional PC model is expressed as follows:

(9) $$PC(x,y)=\frac{\sum_{n}\sum_{\bar{o}}W_{\bar{o}}(x,y)\left\lfloor A_{n\bar{o}}(x,y){\Delta \Phi }_{n\bar{o}}(x,y)-\hat{T}\right\rfloor }{\sum_{n}\sum_{\bar{o}}A_{n\bar{o}}(x,y)+\xi }$$

In Equation (9), $W_{\bar{o}}(x,y)$ is the two-dimensional frequency expansion weight factor; $\hat{T}$ is the noise compensation term; ξ is a small positive value; $\left\lfloor •\right\rfloor$ is an operator that prevents the expression from taking negative values and, when the expression inside the operator is negative, it is set to 0; and ${\Delta \Phi }_{n\bar{o}}(x,y)$ is the two-dimensional phase deviation function.

#### 2.2.2. Noise Estimation

In Equation (9), the noise is assumed to follow the Rayleigh distribution. The noise compensation term is estimated as follows: $T=\mu_{R}+m\sigma_{R}$, where $\mu_{R}$ and $\sigma_{R}$ are the mean and variance of the energy noise, respectively, and m is an empirical parameter. This method suppresses noise to some extent, but multiple parameters in the Rayleigh distribution estimation require empirical setting. Therefore, this study proposes a data-driven method to identify a more accurate T value.

It is demonstrated in [45,46] that the first-order derivatives of various textures also follow the Weibull distribution. The probability density function of the Weibull distribution is expressed as follows:

(10) $$f(x;\lambda ,k)=\left\{\begin{array}{c} \frac{k}{\lambda }{\left(\frac{x}{\lambda }\right)}^{k-1}e^{-{\left(x/\lambda \right)}^{k}},x\geq 0\\ 0,x<0 \end{array}\right.$$

In Equation (10), x is the random variable, λ>0 is the scale parameter, and k>0 is the shape parameter. From the derivation of Equation (10), the mean and variance of the Weibull distribution are given as follows:

(11) $$\left\{\begin{array}{c} E=\lambda \Gamma \left(1+\frac{1}{k}\right)\\ Var=\lambda^{2}\left[\Gamma \left(1+\frac{2}{k}\right)-\Gamma {\left(1+\frac{1}{k}\right)}^{2}\right] \end{array}\right.$$

In Equation (11), λ>0 is the scale parameter, k>0 is the shape parameter, and Γ is the gamma function.

**Figure 4:**
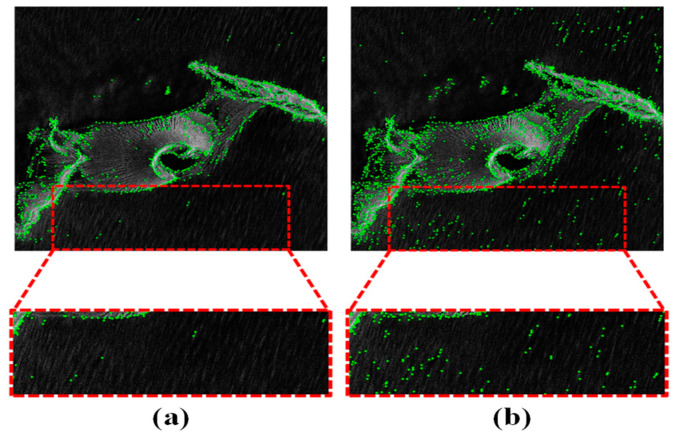
(**a**) The feature detection result using Weibull noise estimation. (**b**) The feature detection result using traditional noise estimation.

First, during the calculation of the PC map, after the multi-directional and multi-scale amplitude responses are summed, the gradient is binned (with the number of bins set to 50 in this paper). A histogram is then computed, and the Weibull distribution can be fitted using the obtained histogram. The mean and variance of the Weibull distribution can be obtained using Equation (11). Finally, the noise compensation term can be estimated as follows:

(12) $$T=\lambda^{2}\left[\Gamma \left(1+\frac{2}{k}\right)-\Gamma {\left(1+\frac{1}{k}\right)}^{2}\right]+k\lambda \Gamma \left(1+\frac{1}{k}\right)$$

where k is a constant. The new noise estimation method has yielded better feature extraction results, as shown in Figure 4.

After noise estimation, a refined PC map can be obtained using Equation (9). According to moment analysis, the axis with the smallest moment is termed the principal axis, which typically contains the directional information of the feature. The largest axis, perpendicular to the principal axis, usually reflects significant feature information. Based on the analysis equations, three intermediate variables should first be calculated:

(13) $$a=\sum_{\theta }{\left(PC(\theta )\cos(\theta )\right)}^{2}$$

(14) $$b=2\sum_{\theta }(PC(\theta )\cos(\theta ))(PC(\theta )\sin(\theta ))$$

(15) $$c=\sum_{\theta }{\left(PC(\theta )\sin(\theta )\right)}^{2}$$

Thus, the formulas for the principal axis ψ and maximum moment $M_{\psi }$ are given as follows:

(16) $$\psi =\frac{1}{2}\arctan(\frac{b}{a-c})$$

(17) $$M_{\psi }=\frac{1}{2}(c+a+\sqrt{b^{2}+{\left(a-c\right)}^{2}})$$

In general, the minimum moment map can be employed to detect “corner points”, while the maximum moment map can be used to detect edge features. In this paper, the maximum moment map is used to detect edge information.

### 2.3. Descriptor Generation

Regarding descriptor generation, the key step is the orientation assignment process. Due to the NRDs in MRSI, conventional gradient-based strategies are prone to orientation reversal problems. In this paper, we utilize the strategy reported in [28] to assign feature orientation based on weighted phase orientations. Here, a weighted bandwidth function for the 2D-Log-Gabor filter is vital to calculate the descriptor:

(18) $$\left\{\begin{array}{c} wc^{\prime }=\overset{6}{\underset{o=1}{\max}}\left(\frac{1}{1+\exp(g\cdot (Cutoff-width_{o}(x,y)))}\right)-\xi \\ wc^{\prime \prime }=\overset{6}{\underset{o=1}{\min}}\left(\frac{1}{1+\exp(g\cdot (Cutoff-width_{o}(x,y)))}\right)-\xi \end{array}\right.$$

In Equation (18), $wc^{\prime }$ and $wc^{\prime \prime }$ represent the maximum and minimum weighting coefficients, respectively; exp is the exponential operator; Cutoff is the fractional measure of frequency extension; $width_{o}(x,y)$ is the fractional image frequency; g controls the sharpness of transitions in the PC; and ξ is the minimum value other than zero.

The maximum and minimum bandwidths of the phase energy components in each orientation and scale are obtained, and the results are then incorporated into the odd-symmetric and even-symmetric functions of the 2D-Log-Gabor filter. This operation effectively mitigates the impact of phase extrema jumps. Therefore, the equation is expressed as follows:

(19) $$WO=\arctan\left(\frac{\sum_{o=1}^{6}\left(wc^{\prime }\cdot \sin\left(\frac{\pi }{6}\cdot o\right)\cdot OO_{o}(x,y)+wc^{\prime \prime }\cdot \sin\left(\frac{\pi }{6}\cdot o\right)\cdot EO_{o}(x,y)\right)}{-\sum_{o=1}^{6}\left(wc^{\prime }\cdot \cos\left(\frac{\pi }{6}\cdot o\right)\cdot OO_{o}(x,y)+wc^{\prime \prime }\cdot \cos\left(\frac{\pi }{6}\cdot o\right)\cdot EO_{o}(x,y)\right)+\Phi }\right)$$

In Equation (19), WO represents the weighted phase orientation feature, $OO_{o}(x,y)$ represents the result of the odd-symmetric convolution after normalization of the o−th layer orientation, and $EO_{o}(x,y)$ represents the normalized even symmetric convolution result of the o−th orientation, $\frac{\pi }{6}\cdot o$ denotes the rotation angle, and Φ is the minimum value (Φ = 0.0001), excluding zero.

Finally, to eliminate the orientation reversal caused by the NRDs, the orientation angles are transformed between $\left[0^{\circ },360^{\circ }\right]$, and the final weighted phase orientation feature is denoted as W, as expressed in the following formula:

(20) $$WP=\left\{\begin{array}{c} WO+\Phi ,WO>0\\ WO+\pi ,WO<0 \end{array}\right.,W=WP⊗\frac{\omega }{\pi }$$

In Equation (20), W represents the final weighted phase orientation feature, WP represents the phase orientation feature in radians, ω is a non-negative constant term (ω=360), and Φ is the minimum value (Φ = 0.0001), excluding zero.

After obtaining the directional features, the principal orientation of the feature points can be calculated. First, the histogram is divided into 36 equal parts, with intervals of 10°, and the phase magnitude and phase orientation features for each part are counted. The maximum peak is identified as the principal orientation. Additionally, peaks with amplitudes greater than 80% of the highest peak are also considered as the principal orientation of the feature points. Using this method, the rotational invariance of features is roughly achieved.

After orientation assignment, this study adopts the MIM strategy for feature description. First, the multi-scale and multi-orientation filtering results $e_{n\bar{o}}(x,y)$, $o_{n\bar{o}}(x,y)$, and $A_{n\bar{o}}(x,y)$ are calculated. Then, for each orientation $\bar{o}$, the magnitudes across all scales are summed to obtain the result $A_{\bar{o}}(x,y)$ over specific orientation $\bar{o}$ as follows:

(21) $$A_{\bar{o}}(x,y)=\sum_{n=1}^{S}A_{n\bar{o}}(x,y)$$

The Log-Gabor convolution layers are arranged sequentially according to the orientations, generating the multi-channel Log-Gabor convolution map ${\left\{A_{\bar{o}}(x,y)\right\}}_{\bar{o}=1}^{O},$ where o represents the number of wavelet orientations. Finally, for each pixel I(x,y), its orientation response sequence is obtained, generating a vector with o dimensions. The maximum value of this o-dimensional array is then calculated, and the index corresponding to this maximum value is used as the value at the same pixel location in the MIM.

After generating the final maximum index map, the MIM around the feature point is rotated according to the feature orientation calculated above to obtain the descriptor required in this paper.

### 2.4. Cascade Matching Guided by Global Position, Orientation, and Scale Information

In addition to significant NRDs, MRSI is often affected by scale and orientation differences. To mitigate them, this study proposes a cascade matching strategy based on the initial matching results and global position, orientation, and scale guidance. First, feature points are detected, and corresponding descriptors are generated at each scale space layer. Then, global matching is performed on the entire scale space. It should be noted that since the scale space in this paper is not downsampled, the matching time will increase exponentially with the increase in the number of scale space layers. Therefore, the number of scale space layers should be limited (the empirical value is 3 layers). Finally, the fast sample consensus (FSC) algorithm [47] is used to further refine the matching and obtain the coarse affine transformation matrix H.

Affine transformation is a linear mapping method that preserves points, straight lines, and planes. It is commonly used to correct geometric distortions, including translation, rotation, scaling, and shearing transformations. Therefore, for the affine transformation matrix H, we have the following expression:

(22) $$\left[\begin{array}{ccc} a & c & e\\ b & d & f\\ 0 & 0 & 1 \end{array}\right]=\left[\begin{array}{ccc} 1 & 0 & e\\ 0 & 1 & f\\ 0 & 0 & 1 \end{array}\right]*\left[\begin{array}{ccc} \cos\theta_{u} & -\sin\theta_{u} & 0\\ \sin\theta_{u} & \cos\theta_{u} & 0\\ 0 & 0 & 1 \end{array}\right]*\left[\begin{array}{ccc} S_{x} & 0 & 0\\ 0 & S_{y} & 0\\ 0 & 0 & 1 \end{array}\right]*\left[\begin{array}{ccc} 1 & sh_{y} & 0\\ sh_{x} & 1 & 0\\ 0 & 0 & 1 \end{array}\right]$$

In Equation (22), $\theta_{u}$ is the rotation angle, $S_{x}$ represents the scale difference in the x-orientation, $S_{y}$ represents the scale difference in the y-orientation, $sh_{x}$ is the shear factor along the x-axis, and $sh_{y}$ is the shear factor along the y-axis.

By applying the transformation to Equation (22), the following system of equations is obtained:

(23) $$\left\{\begin{array}{c} a=S_{x}*\cos\theta_{u}-sh_{x}*S_{y}*\sin\theta_{u}\\ b=S_{x}*\sin\theta_{u}+sh_{x}*S_{y}*\cos\theta_{u}\\ c=sh_{y}*S_{x}*\cos\theta_{u}-S_{y}*\sin\theta_{u}\\ d=sh_{y}*S_{x}*\sin\theta_{u}+S_{y}*\cos\theta_{u} \end{array}\right.$$

Finally, the rotation angle $\theta_{u}$ and the scaling difference $S_{scale}$ of the image can be obtained from the above system of equations:

(24) $$\left\{\begin{array}{c} \theta_{u}=\arctan\left(\frac{b}{a}\right)\\ S_{x}=\sqrt{a^{2}+b^{2}}\\ S_{y}=\frac{a^{*}d-b^{*}c}{\sqrt{a^{2}+b^{2}}}\\ S_{scale}=\sqrt{S_{x}^{2}+S_{y}^{2}} \end{array}\right.$$

Based on the affine transformation matrix H, the rotation angle $\theta_{u}$ is calculated using Equation (24). This relative angle represents the angle difference between the images to be matched. For simplicity, we assume that the principal orientation of the reference image is $0^{\circ }$, then the principal orientation of the target image is $\theta_{u}$. By adjusting the principal orientation of all feature points in the two images, the influence of the principal orientation estimation on the matching performance is eliminated. Similarly, given matrix H, the scaling difference $S_{scale}$ can be calculated using Equation (24). Then, the radius of the descriptor is adjusted using $S_{scale}$. Finally, the descriptor is regenerated using the refined principal orientation and descriptor radius. Since the computational complexity of the MIM descriptor is very low, the whole process is very fast. After the descriptor is regenerated, it is necessary to limit the matching range to further improve the matching accuracy. Starting from matrix H, it is easy to map each feature point from the reference image to the target image and then match it only with the K closest points in the target image (K is an empirical value, generally not less than 10, and set to 20 in this paper). Finally, the FSC algorithm is executed again to eliminate mismatches and output the matching results.

## 3. Experimental Results and Discussion

In this section, we validate the effectiveness of the proposed registration method by comparing it with eight state-of-the-art algorithms, including six feature-based methods—namely, PSO-SIFT [22], LGHD [48], RIFT [11], HAPCG [27], COFSM [23], and RIFT2—as well as two deep learning-based methods: CMM-Net [49] and RedFeat [50]. The experimental datasets, parameters, and results are presented in detail below.

### 3.1. Experimental Setting

#### 3.1.1. Image Datasets

Three multi-modal datasets were selected to evaluate the method. Dataset 1 contains six modalities of remote sensing image pairs. These include optical–optical images with variable time differences, optical–SAR images, optical–map images, optical–infrared images, optical–depth images, and day–night images. These cover almost all application scenarios of MRSI matching. Each type contains 10 image pairs, most of which have significant NRDs and are highly representative. Dataset 2 contains five modalities of remote sensing image pairs, including depth–optical, infrared–optical, map–optical, SAR–optical, and night–day images. Each type includes 10 image pairs. These image pairs not only exhibit NRDs but also include geometric transformations such as scale variations, rotations, and displacements. Dataset 3 consists of 477 RGB–NIR image pairs with nine scenes, including country, field, forest, indoor, mountains, old buildings, street, urban, and water, which are taken from reference [51]. Detailed information about these three datasets is provided in Table 1 below.

#### 3.1.2. Evaluation Criteria

In this study, the matching performance is analyzed using three criteria: NCM, RCM, and RMSE. If the Euclidean distance between a pair of points satisfies the nearest neighbor distance ratio (NNDR) constraint, then this pair of points will be termed a match. A correct correspondence means a match where two points correspond to the same physical location in two images. The NCM is obtained by counting the number of such correspondences. RCM is defined as follows:

(25) $$RCM=\frac{N_{C}}{N_{C}+N_{F}}$$

where $N_{C}$ denotes the number of correct correspondences, and $N_{F}$ represents the number of false correspondences.

The RMSE of correct matches is defined as follows:

(26) $$RMSE=\sqrt{\frac{1}{N}\sum_{i=1}^{N}{\left(x_{i}^{\prime }-x_{i}^{\prime \prime }\right)}^{2}+{\left(y_{i}^{\prime }-y_{i}^{\prime \prime }\right)}^{2}}$$

where N represents the number of correct matching points, $\left(x_{i}^{\prime },y_{i}^{\prime }\right)$ denotes the coordinate of the matched point transformed by estimated affine matrix, and $\left(x_{i}^{\prime \prime },y_{i}^{\prime \prime }\right)$ denotes its matching point in the reference image.

#### 3.1.3. Experimental Environments

In the paper, the hand-crafted methods are implemented in MATLAB R2021b, and the deep learning-based methods are implemented using Python 3.9. The experiments were conducted on a Windows 11 laptop equipped with an Intel(R) Core(TM) i5-1240P CPU at 1.70 GHz and an Intel(R) Iris(R) Xe Graphics 128 MB GPU.

### 3.2. Parameter Analysis

Our proposed method contains three main steps: the construction of the side window scale space, feature extraction and descriptor generation, and the global position-, orientation-, and scale-guided cascade matching. Three parameters are analyzed, as shown in Table 2, and the other parameters follow the definitions set by the author in previous work.

The parameter r represents the filter window radius of SWF. In general, the larger the radius, the stronger the filtering effect. The parameter $N_{L}$ represents the number of layers in the side window scale space. Generally, the more layers, the more feature points can be extracted, but the time consumed will also be higher. The parameter K represents the number of feature points to be searched. In the cascade matching process, the initial affine transformation matrix H is used to match the feature points in the reference image with the corresponding points in the target image. If K is too small, the optimal point may not be found. If K is too large, the matching time cost will increase.

This section introduces parameter studies based on 60 pairs of MRSI. Three groups of independent experiments were designed to determine the parameters r, $N_{L}$, and K, where each experiment has only one parameter as a variable, with the other parameters remaining constant. For each parameter, RMSE, NCM, and RCM are used as evaluation metrics. The detailed summary of the experimental settings is provided in Table 2, Table 3, Table 4 and Table 5.

The following conclusions can be drawn from the experimental results. The larger the parameter r, the stronger the smoothing effect of SWF and the faster the filtering speed. However, it may be too large to lose some edge information. From Table 3, when the parameters $N_{L}$ and K remain unchanged and r=6, the performance is best, and both RMSE and NCM are optimal under this setting. Therefore, r is set to 6 in the subsequent experiments. From Table 4, comprehensively considering RMSE and RCM, K is set to 20 in this study. From Table 5, the best performance is achieved when $N_{L}=3$. If $N_{L}$ is too large, the time consumed will increase rapidly, and the matching performance will decrease. Comprehensively considering RMSE, NCM, and RCM, $N_{L}$ is set to 3 in this paper.

### 3.3. Matching Results Analysis

#### 3.3.1. Qualitative Analysis

**Figure 5:**
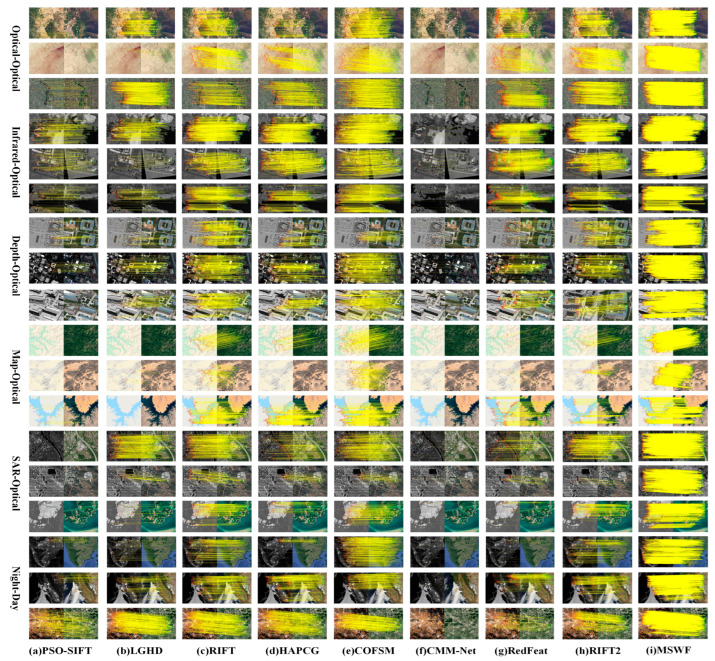
Matching results of the COFSM dataset using nine methods.

**Figure 6:**
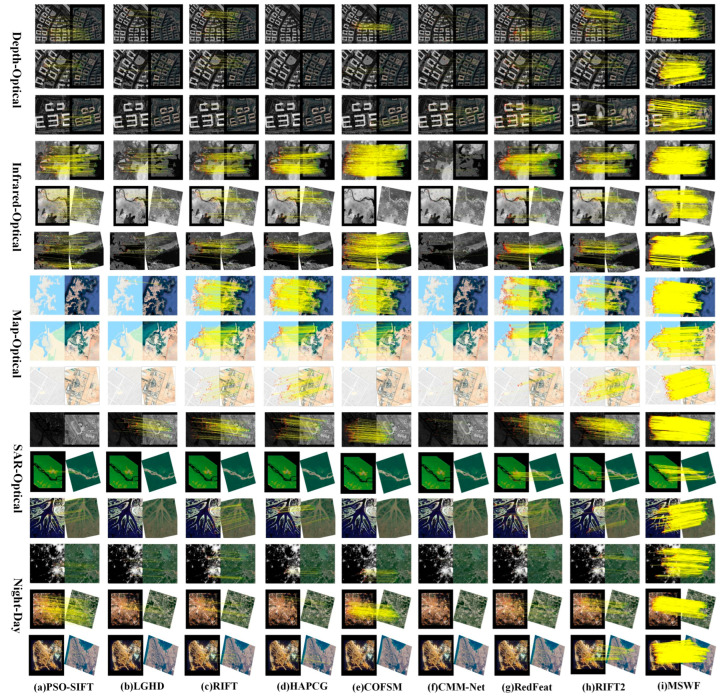
Matching results of the HOWP dataset using nine methods.

**Figure 7:**
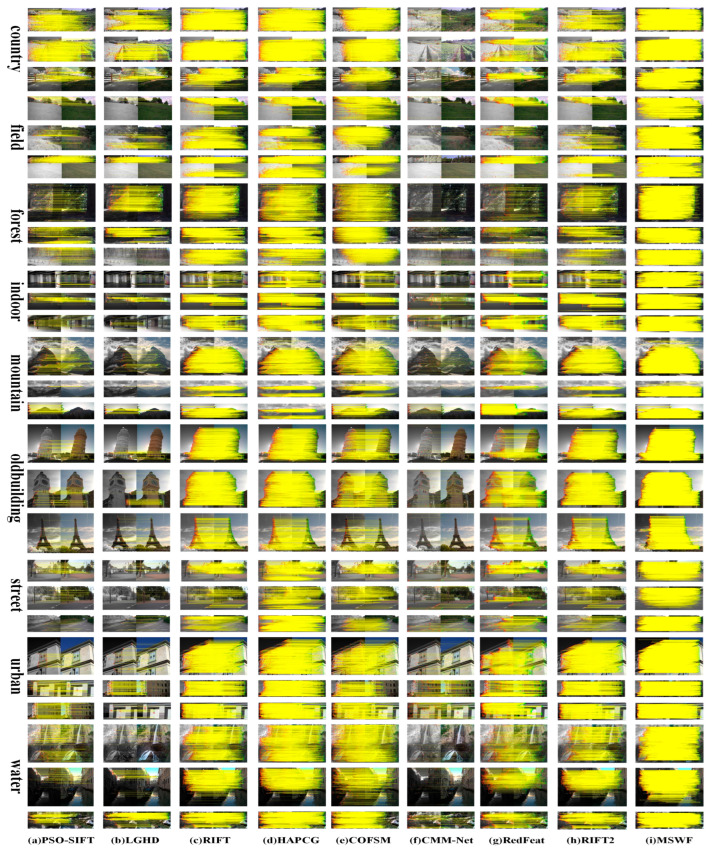
Matching results of the RGB-NIR dataset using nine methods.

This study selects typical image pairs and matching results from three datasets, as shown in Figure 5, Figure 6 and Figure 7.

In Figure 5, the matching accuracy of PSO-SIFT is poor, with an average RMSE of 4.3. PSO-SIFT extracts image features by calculating the second-order gradient. This method is sensitive to NRDs and noise. The LGHD method uses image frequency domain features for matching. The matching effect is relatively good in optical–optical, optical–infrared, and optical–map modes (rows 1, 3, 4, 5, and 6), but this method cannot effectively handle scale and rotation differences (row 5). HAPCG is better than the first two methods in processing optical–optical, optical–infrared, and optical–map modes (rows 1 to 9). However, the multi-directional convolution response of the symmetric filter will cause orientation reversal. Thus, HAPCG is unable to obtain the correct orientation information, resulting in a decrease in matching performance (rows 10, 11, 13, 14, 15, and 16). The RIFT method performs poorly in optical–SAR and day–night modes, probably because RIFT is not scale-invariant. The COFSM method performs relatively well overall on the dataset. However, in the SAR–optical section (row 15), the number of successfully matched points is small, with an average NCM of 158.7 and an average RMSE of 4.89. The matching results of CMM-Net and RedFeat are not ideal (rows 10 to 18), which shows that deep learning methods have limitations. The RIFT2 method performs relatively well on the dataset overall. However, in the map–optical mode, the number of successfully matched points is relatively small, with an average NCM of 102.67 and an average RMSE of 2.57. Obviously, MSWF has the largest number of correctly matched points among all methods, with an average NCM of 875, far exceeding the other eight best methods. This value is 2.96 times better than COFSM and 3.74 times better than RIFT. The average RMSE of MSWF is 2.13, the lowest in the entire dataset, representing a 37.3% decrease compared to COFSM. The average RCM is 81.6%. This value is 22.0% and 33.1% higher than the two methods with the best average RCM—namely, RedFeat and COFSM, respectively.

From Figure 6, PSO-SIFT fails to match most of the images in the optical–map and optical–SAR modes (rows 7, 8, 9, 10, and 11), with an average NCM of only 29.2 and an average RMSE of 7.34. The LGHD and RIFT methods have good matching results, but their ability to handle scale and rotation differences is poor. HAPCG performs poorly when dealing with image pairs with rotation differences, and even fails to match (rows 5, 9, 12, and 14). Compared with manual methods, RedFeat can successfully match in most cases. However, when the image rotation angle exceeds 30°, its performance drops sharply (row 14). COFSM performs better than previous methods when dealing with smaller rotations, translations, and scale changes, with an average RMSE of 3.88, an average NCM of 147.28, and an average RCM of 0.38 for the entire dataset. The RIFT2 method has relatively good matching results but only in the map–optical mode, with an average RMSE of 4.48, while the matching results in the other modes are not ideal.

The MSWF method shows the best performance, demonstrating the best results when dealing with large scale differences (rows 1, 2, and 3), rotations (rows 5, 6, 9, 12, and 14), and significant NRD differences (rows 4, 7, 8, 10, and 15). Its average NCM is 407.8, which is 176.8% higher than COFSM. The average RMSE is 2.94, which is 24.2% lower than COFSM, and the average RCM is 0.48, which is 23.3% higher than COFSM.

From Figure 7, PSO-SIFT has poor matching results in field, forest, and water scenes because the gradient features in these scenes are not obvious. The three manual methods RIFT, HAPCG, and COFSM all show good matching results, but their performance is slightly worse in field and street scenes. The RIFT2 method performs relatively well on the entire dataset, with an average NCM of 946.69. MSWF has the best performance, with an average RMSE of 1.38. Compared with other methods, the average RMSE is reduced by 39.1%~89.1%. In forest, indoor, old building, and urban scenes, the average RMSE is less than one pixel, which has an absolute advantage over other methods. The average RCM is 88.9%, which is 10.2% higher than other methods, reaching 388.9%. The average NCM is 2579, which is only lower than the HAPCG method. This is because MSWF limits the maximum number of feature points per layer in the scale space to 5000 to ensure robustness, while HAPCG does not impose such a restriction, resulting in a lower number of correct matches for MSWF than HAPCG. However, compared with other methods, the average NCM of MSWF increased by 49% to 2356.2%.

**Figure 8:**
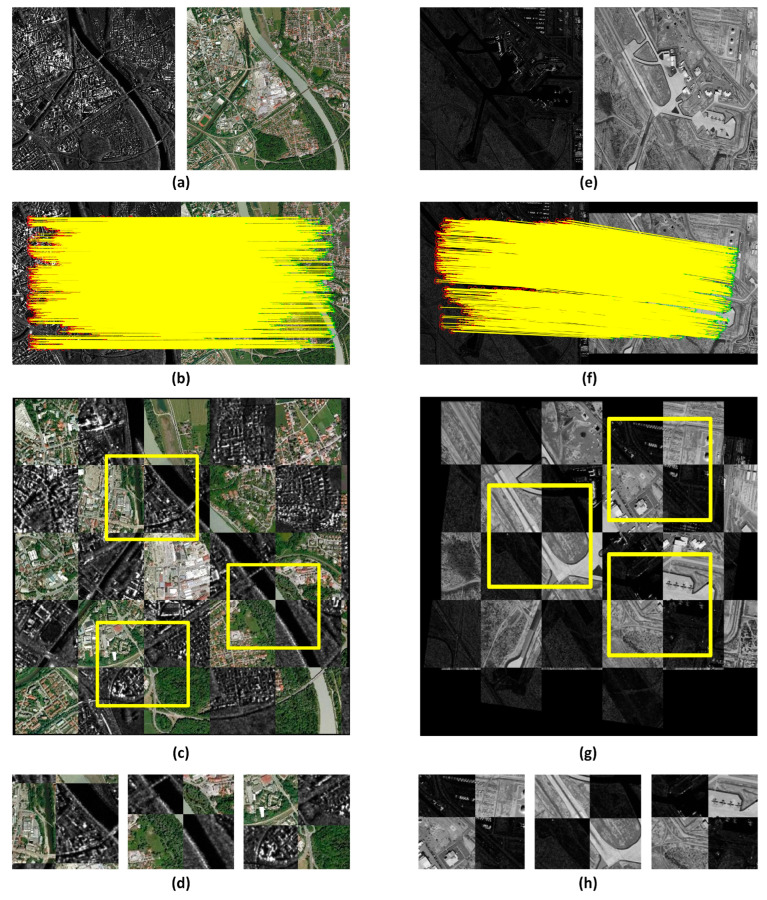
Registration results of image pairs in COFSM and HOWP datasets. (**a**,**e**) Input images. (**b**,**f**) corresponding key points. (**c**,**g**) Fusion result. (**d**,**h**) Enlarged sub-images.

**Figure 9:**
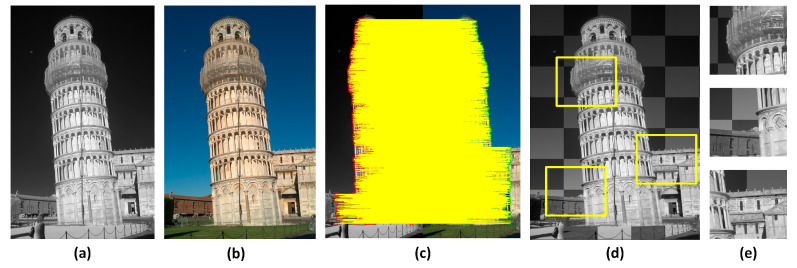
Registration results of image pairs in RGB-NIR datasets. (**a**,**b**) Input images. (**c**) Corresponding key points. (**d**) Fusion result. (**e**) Enlarged sub-images.

To more clearly demonstrate the matching effect of our method, we selected the matching results of different images from the three datasets and provided local enlarged images as shown in Figure 8 and Figure 9.

The extensive experiments above demonstrate that the proposed MSWF method achieves the most robust matching for images with NRDs. It also shows great potential in addressing scale, rotation, and displacement differences. Its outstanding performance is attributed to the constructed side window scale space, effective noise estimation, weighted phase orientation feature, and the global position-, orientation-, and scale-guided cascade matching strategy.

#### 3.3.2. Quantitative Analysis

**Figure 10:**
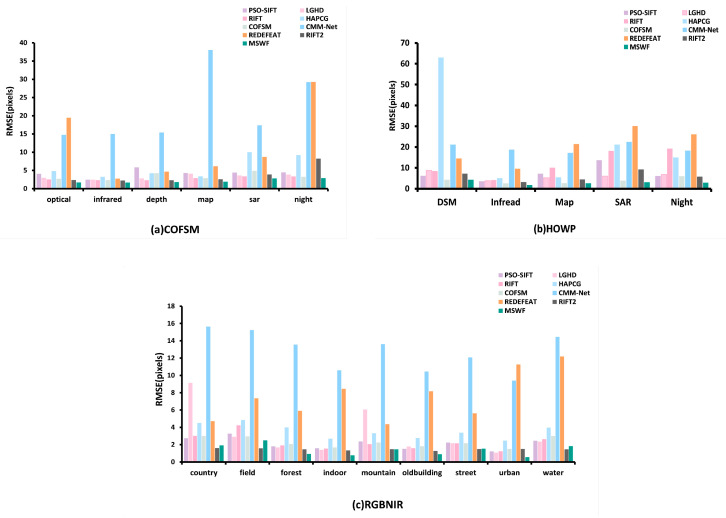
Matching accuracy of nine methods on three datasets.

Figure 10 shows the RMSE performance results of the proposed method compared to the other eight methods across three datasets. MSWF achieves the lowest RMSE results in all image datasets.

In the COFSM dataset, the matching performance of PSO-SIFT and LGHD are both poor. COFSM performs poorly on optical–depth and optical–SAR modes, with average RMSE values of 4.27 and 4.89, respectively. The average RMSE of the RIFT2 method on the entire dataset is 3.60, which is still not ideal. Compared with previous methods, RIFT performs better, with an average RMSE of 2.80. RedFeat performs relatively well only on optical–infrared images, with an average RMSE of 2.73. The MSWF method proposed in this study shows the best performance, with an average RMSE of 2.13, which is 37.3%~90.16% lower than other methods.

In the HOWP dataset, HAPCG performs poorly in most modalities. The MIM descriptor constructed by RIFT has a significant performance degradation when dealing with large-scale differences and large rotations, with an average RMSE of 12.01. The average RMSE values of the two deep learning methods, CMM-Net and RedFeat, were 20.35 and 19.58, respectively, revealing the limitations of deep learning methods in processing MRSI. COFSM performed relatively well, with an average RMSE of 3.88. MSWF had the best matching accuracy, with an average RMSE of 2.94, which was 24.3%~86.6% lower than other methods.

In the RGB-NIR dataset, RIFT and COFSM have similar matching accuracy on the RGB-NIR dataset, with average RMSE values of 2.27 and 2.28, respectively. The RIFT2 method has a better matching effect in the field scene, with an average RMSE of 1.57. RedFeat performs relatively well in country and mountain scenes. Compared with the above methods, MSWF has the best matching accuracy, with an average RMSE of 1.38 for the entire RGB-NIR dataset. It is the only method among the nine methods with an RMSE less than 1.4 pixels, and its RMSE is reduced by 39.0%~89.1% compared with other methods.

**Figure 11:**
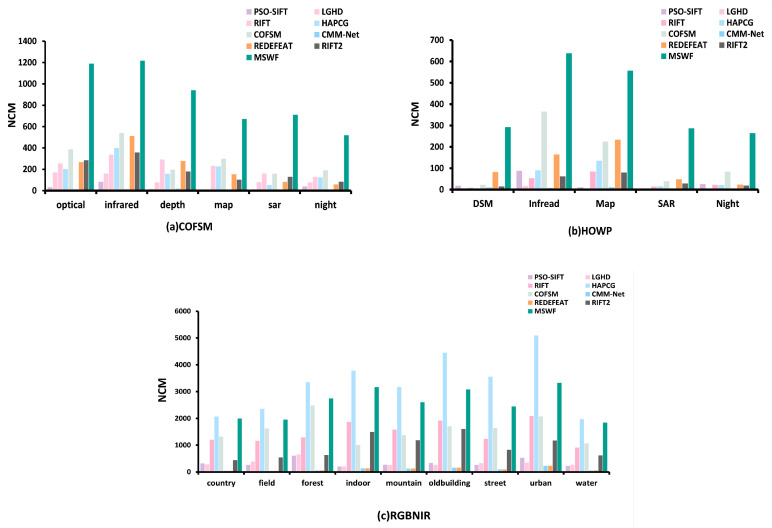
Matching NCM of nine methods on three datasets.

Figure 11 presents the quantitative comparison results of the nine methods based on the NCM metric across three datasets.

In the COFSM dataset, PSO-SIFT and LGHD performed poorly overall, with average NCM values of 233.95 and 225.53 for RIFT and RedFeat, respectively. The average NCM of the RIFT2 method is 189.38. COFSM performed well overall, with an average NCM of 295.3. MSWF matched the most point pairs, with an average NCM of 710.6 for the SAR-optical, which was at least 347.7% higher than other methods. MSWF was the only method that did not show a significant performance drop in the SAR–optical mode. The average NCM of the entire COFSM dataset was 875.1, which was at least 196.3% higher than other methods.

In the HOWP dataset, PSO-SIFT, LGHD, RIFT, and CMM-Net RIFT2 performed poorly in almost all modalities, with average NCMs of 29.24, 6.24, 36.7, 7.3, and 40.8, respectively. RedFeat and COFSM only achieved good results in optical–infrared and optical–map images, with average NCM values of 110.34 and 147.28, respectively. MSWF performed the most robustly, with an average NCM of 407.78 for the entire dataset—at least 176.8% higher.

In the RGB-NIR dataset, the average NCM of the RedFeat method was 837.86. The average NCM of the RIFT2 method is 946.69. The average NCM values of RIFT and COFSM were 1473.43 and 1589.96, respectively, and the average NCM of MSWF on the entire dataset was 2579.2. This is lower than the average NCM of HAPCG of 3311.97. Given that MSWF limits the number of feature points in each layer in the scale space to ensure robustness, its RMSE and RCM are higher than those of HAPCG; however, the NCM of MSWF is lower than that of HAPCG. If HAPCG is excluded, the average NCM of MSWF is at least 62.2% higher than that of other methods.

**Figure 12:**
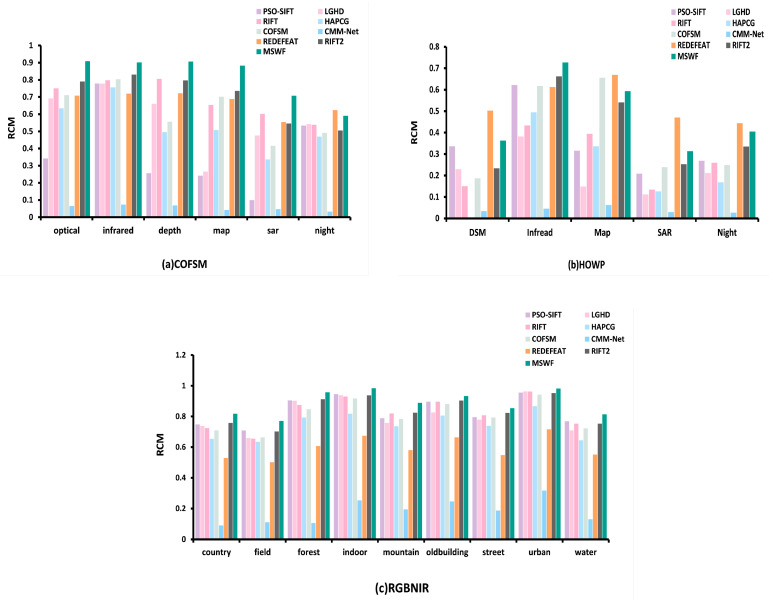
Matching RCM of nine methods on three datasets.

Figure 12 shows the quantitative comparison results of the nine methods based on the RCM metric across three datasets.

In the COFSM dataset, LGHD and HAPCG show similar RCM values, with average values of 56.8% and 53%, respectively. COFSM has an average RCM of 61.3%, which is slightly improved compared with the previous methods. RIFT and RedFeat methods show good performance, with average RCM values of 69.14% and 66.93%, respectively, and perform well on the entire dataset. The average RCM of the RIFT2 method is 70.07%. MSWF has the highest RCM and is the only method among the nine methods with an RCM greater than 80%. The average RCM for MSWF reaches 81.6%, which is much higher than other methods.

In the HOWP dataset, since PSO-SIFT has fewer correctly matched points and the obtained RCM is inaccurate, we do not consider the PSO-SIFT results. HAPCG has an average RCM of only 0.37% in the optical–deep mode, almost completely failing to match. The COFSM and RIFT2 methods performed relatively well, with average RCM values of 38.92% and 40.5%, respectively. MSWF has an average RCM of 48.02%, which is better than COFSM and second only to the deep learning-based RedFeat method.

In the RGB-NIR dataset, LGHD obtains an RCM of 80.8% on the entire dataset. HAPCG performs poorly with an average RCM of 74.34%. RedFeat has an average RCM of 59.7%. The RIFT2 method performs relatively well, with an average RCM of 84.06%. MSWF has the highest RCM and is the only method with an average RCM greater than 85%, with an average RCM of 88.88%.

#### 3.3.3. Analysis of Rotation Invariance

**Figure 13:**
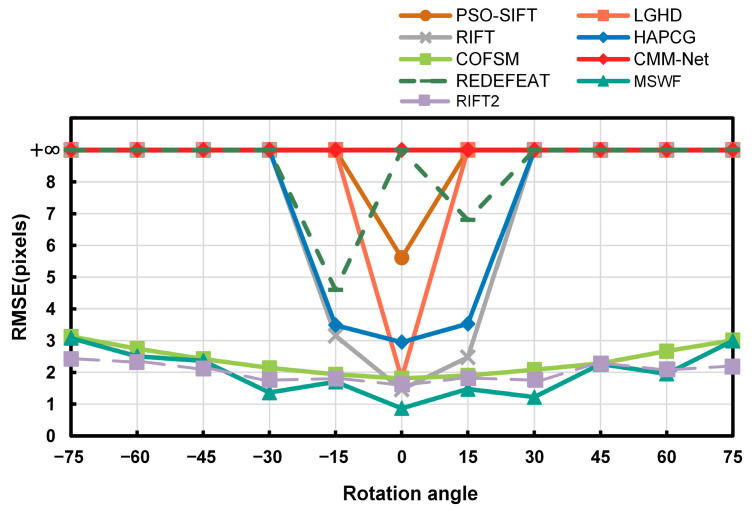
RMSE results of 20 sets of pictures at multiple angles.

To validate the rotational invariance of MSWF [40], twenty image pairs were selected from the RGB-NIR dataset for testing. For each image pair, the reference image was rotated clockwise and counterclockwise in 15 º intervals, generating five simulated images in each orientation. This resulted in ten sets of simulated image pairs for matching tests. The RMSE metric was compared with the other seven methods, and the corresponding results are shown in Figure 13 (here, the symbol, +∞ represents pairs where matching failed or RMSE>9). Figure 13 shows that, except for the COFSM and RIFT2 methods, the rotational invariance of the other six methods is poor, especially for large rotations ($\left|rotation\right|\geq 30^{\circ }$), where almost all six methods fail to match or have RMSE>9. The rotational invariance of the COFSM method is relatively good, but still not sufficient.

The rotation invariance of the MSWF method proposed in this paper is close to the performance of the RIFT2 method and is better than the RIFT2 method in a small range of rotation ($\left|rotation\right|\leq 30^{\circ }$), showing the best rotation invariance. This is because the weighted phase-directional features better preserve the shape components of the image, effectively mitigating issues such as phase orientation inversion and abrupt phase extrema, thereby providing a better estimation of the principal orientation.

## 4. Discussion

As discussed above, the experiments detailed in this paper were performed on three datasets, and the proposed MSWF method achieved the best performance in terms of RMSE, NCM, and RCM (Figure 5, Figure 6, Figure 7, Figure 8, Figure 9 and Figure 10). This performance is due to its three elaborately designed constituents. The constructed side window scale space preserves contour information and increases the number of feature points extracted from most modalities. In the feature descriptor generation stage, the weighted phase orientation feature has a more robust orientation alignment strategy, thereby increasing its rotational invariance, as demonstrated in Figure 11. Finally, the global-to-local cascade matching strategy not only refines the matching results but also retrieves the eliminated correct correspondences, further improve the matching performance.

To further illustrate the effectiveness of the global position-, orientation-, and scale-guided matching strategy proposed in this paper, 60 pairs of images were selected under six modalities to show the results of coarse matching and fine matching stages to prove the effectiveness of the strategy. These results are shown in Table 6.

## 5. Conclusions

This study proposed a new robust matching method, termed MSWF, that can effectively mitigate NRDs and geometric distortions in MRSI. First, a side window scale space is constructed to better preserve the edge information of the images. Then, a redefined noise estimation method is proposed, which allows for the extraction of more robust feature points. Subsequently, an improved weighted phase orientation feature is introduced to effectively address issues such as phase orientation inversion in MRSI matching. After obtaining the principal orientation, the MIM descriptor for that orientation is generated for initial global matching. Finally, based on the initial matching information, a position-, orientation-, and scale-guided cascade matching strategy is applied to obtain the final optimal matching results. Experimental results demonstrated that MSWF outperforms state-of-the-art methods such as PSO-SIFT, LGHD, RIFT, RIFT2, HAPCG, COFSM, CMM-Net, and RedFeat in terms of RMSE, NCM, and RCM.

The main deficiency of the proposed MSWF is that the matching performance may degrade when there are significant scale differences between images. In future work, we aim to address this issue by improving the construction of the scale space to enhance the matching robustness at different scales.

## Figures and Tables

**Table 1:** The detailed information of the experimental datasets.

Dataset Name	Modalities	Size	Source
COFSM	Optical, infrared, depth, map, SAR, day, night	60	https://skyearth.org/publication/project/CoFSM/
HOWP	Optical, depth, infrared, map SAR, day, night	50	https://skyearth.org/publication/project/HOWP/
RGB-NIR	Optical, NIR	477	https://www.epfl.ch/labs/ivrl/research/downloads/rgb-nir-scene-dataset/

**Table 2:** The parameter settings.

Experiments	Variable	Fixed Parameters
Parameter r	$r=\left[2,4,6,8,10\right]$	$N_{L}=3,K=20$
$\mathrm{Parameter}N_{L}$	$N_{L}=\left[1,2,3,4\right]$	r=6,K=20
Parameter K	$K=\left[10,20,30,40,50\right]$	$r=6,N_{L}=3$

**Table 3:** The results of parameter r.

Metric	$N_{L}=3,K=20$				
	**2**	**4**	**6**	**8**	**10**
RMSE	2.23	2.27	2.13	2.242	2.247
NCM	815.3	819.2	875.09	826.23	821.65
RCM	80.5.%	80.08%	81.6%	81.8%	82.0%

**Table 4:** The results of parameter K.

Metric	$r=6,N_{L}=3$				
	**10**	**20**	**30**	**40**	**50**
RMSE	2.26	2.15	2.30	2.44	2.22
NCM	870.05	841.72	809.78	806.10	804.50
RCM	82.6.%	83.9%	82.1%	81.3%	81.0%

**Table 5:** The results of parameter $N_{L}$.

Metric	r=6,K=20			
	**1**	**2**	**3**	**4**
RMSE	2.34	2.29	2.15	2.25
NCM	829.56	839.16	841.72	818.41
RCM	81.5%	81.78%	83.9%	82.28%

**Table 6:** Comparison of coarse matching and fine matching results.

Modalities		Optical	Infrared	Depth	Map	SAR	Day–Night
RMSE	Initial	2.82	2.33	2.63	2.44	3.76	3.62
	Improved	1.69	1.66	1.81	1.90	2.81	2.87
NCM	Initial	553.05	762.83	342.48	408.32	424.63	358.67
	Improved	1189.67	1217.88	941.3	671.67	710.6	519.45
RCM	Initial	71.7%	80.9%	71.8%	81.3%	54.9%	46.2%
	Improved	90.9%	90.2%	90.7%	88.3%	70.7%	59.0%

## Data Availability

The original data presented in the study are openly available; all the URLs are listed in the paper.

## References

[1] Song Y., He Z., Qian H., Du X. (2023). Vision transformers for single image dehazing. IEEE Trans. Image Process..

[2] Li X., Shao H., Lu S., Xiang J., Cai B. (2022). Highly efficient fault diagnosis of rotating machinery under time-varying speeds using LSISMM and small infrared thermal images. IEEE Trans. Syst. Man Cybern. Syst..

[3] Zhang Y., Liu Y., Zhang H., Ma G. (2022). Multimodal remote sensing image matching combining learning features and Delaunay triangulation. IEEE Trans. Geosci. Remote Sens..

[4] Yi H., Liu B., Zhao B., Liu E. (2024). LiDAR-Guided Stereo Matching Using Bayesian Optimization with Gaussian Process Regression. IEEE Geosci. Remote Sens. Lett..

[5] Ye Y., Shan J., Hao S., Bruzzone L., Qin Y. (2018). A local phase based invariant feature for remote sensing image matching. ISPRS J. Photogramm. Remote Sens..

[6] Zitova B., Flusser J. (2003). Image registration methods: A survey. Image Vis. Comput..

[7] Ma J., Ma Y., Li C. (2019). Infrared and visible image fusion methods and applications: A survey. Inf. Fusion.

[8] Jiang X., Ma J., Xiao G., Shao Z., Guo X. (2021). A review of multimodal image matching: Methods and applications. Inf. Fusion.

[9] Ma J., Jiang X., Fan A., Jiang J., Yan J. (2021). Image Matching from Handcrafted to Deep Features: A Survey. Int. J. Comput. Vis..

[10] Ye Y., Zhu B., Tang T., Yang C., Xu Q., Zhang G. (2022). A robust multimodal remote sensing image registration method and system using steerable filters with first- and second-order gradients. ISPRS J. Photogramm. Remote Sens..

[11] Anandan P. (1987). Measuring Visual Motion from Image Sequences.

[12] Kai B., Uwe D.H. (2001). Template matching using fast normalized cross correlation. Opt. Pattern Recognit. XII.

[13] Chen H.M., Arora M.K., Varshney P.K. (2003). Mutual information-based image registration for remote sensing data. Int. J. Remote Sens..

[14] Ye Y., Shan J. (2014). A local descriptor based registration method for multispectral remote sensing images with non-linear intensity differences. ISPRS J. Photogramm. Remote Sens..

[15] Ye Y., Shen L., Hao M., Wang J., Xu Z. (2017). Robust Optical-to-SAR Image Matching Based on Shape Properties. IEEE Geosci. Remote Sens. Lett..

[16] Ye Y., Shen L. (2016). Hopc: A novel similarity metric based on geometric structural properties for multi-modal remote sensing image matching. ISPRS Ann. Photogramm. Remote Sens. Spat. Inf. Sci..

[17] Xiong X., Xu Q., Jin G., Zhang H., Gao X. (2020). Rank-Based Local Self-Similarity Descriptor for Optical-to-SAR Image Matching. IEEE Geosci. Remote Sens. Lett..

[18] Ye Y., Bruzzone L., Shan J., Bovolo F., Zhu Q. (2019). Fast and Robust Matching for Multimodal Remote Sensing Image Registration. IEEE Trans. Geosci. Remote Sens..

[19] Lowe D.G. (2004). Distinctive Image Features from Scale-Invariant Keypoints. Int. J. Comput. Vis..

[20] Dellinger F., Delon J., Gousseau Y., Michel J., Tupin F. (2015). SAR-SIFT: A SIFT-Like Algorithm for SAR Images. IEEE Trans. Geosci. Remote Sens..

[21] Xiang Y., Wang F., You H.J. (2018). OS-SIFT: A robust SIFT-like algorithm for high-resolution optical-to-SAR image registration in suburban areas. IEEE Trans. Geosci. Remote Sens..

[22] Ma W., Wen Z., Wu Y., Jiao L., Gong M., Zheng Y., Liu L. (2017). Remote Sensing Image Registration With Modified SIFT and Enhanced Feature Matching. IEEE Geosci. Remote Sens. Lett..

[23] Yao Y., Zhang Y., Wan Y., Liu X., Yan X., Li J. (2022). Multi-Modal Remote Sensing Image Matching Considering Co-Occurrence Filter. IEEE Trans. Image Process..

[24] Kovesi P. (1999). Image features from phase congruency. J. Comput. Vis. Res..

[25] Kovesi P. (2000). Phase congruency: A low-level image invariant. Psychol. Res..

[26] Li J., Hu Q., Ai M. (2020). RIFT: Multi-Modal Image Matching Based on Radiation-Variation Insensitive Feature Transform. IEEE Trans. Image Process..

[27] Yao Y., Zhang Y., Wan Y., Liu X., Guo H. (2021). Heterologous Images Matching Considering Anisotropic Weighted Moment and Absolute Phase Orientation. Geomat. Inf. Sci. Wuhan Univ..

[28] Zhang Y., Yao Y., Wan Y., Liu W., Yang W., Zheng Z., Xiao R. (2023). Histogram of the orientation of the weighted phase descriptor for multi-modal remote sensing image matching. ISPRS J. Photogramm. Remote Sens..

[29] Hou Z., Liu Y., Zhang L. (2024). POS-GIFT: A geometric and intensity-invariant feature transformation for multimodal images. Inf. Fusion.

[30] Chen J., Chen X., Chen S., Liu Y., Rao Y., Yang Y., Wang H., Wu D. (2023). Shape-Former: Bridging CNN and Transformer via ShapeConv for multimodal image matching. Inf. Fusion.

[31] Ye F., Su Y., Xiao H., Zhao X., Min W. (2018). Remote sensing image registration using convolutional neural network features. IEEE Geosci. Remote Sens. Lett..

[32] Ma W., Zhang J., Wu Y., Jiao L., Zhu H., Zhao W. (2019). A novel two-step registration method for remote sensing images based on deep and local features. IEEE Trans. Geosci. Remote Sens..

[33] Hughes L.H., Marcos D., Lobry S., Tuia D., Schmitt M., Sensing R. (2020). A deep learning framework for matching of SAR and optical imagery. ISPRS J. Photogramm. Remote Sens..

[34] Wang Q., Zhang J., Yang K., Peng K., Stiefelhagen R. (2022). Matchformer: Interleaving attention in transformers for feature matching. Lecture Notes in Computer Science.

[35] Xie H., Zhang Y., Qiu J., Zhai X., Liu X., Yang Y., Zhao S., Luo Y., Zhong J.J.I.F. (2023). Semantics lead all: Towards unified image registration and fusion from a semantic perspective. Inf. Fusion.

[36] Yang C., Gong G., Liu C., Deng J., Ye Y. (2025). RMSO-ConvNeXt: A Lightweight CNN Network for Robust SAR and Optical Image Matching Under Strong Noise Interference. IEEE Trans. Geosci. Remote Sens..

[37] Zhu B., Ye Y., Dai J., Peng T., Deng J., Zhu Q. (2024). VDFT: Robust feature matching of aerial and ground images using viewpoint-invariant deformable feature transformation. ISPRS J. Photogramm. Remote Sens..

[38] Lin S., Huang F., Lai T., Lai J., Wang H., Weng J. (2024). Robust Heterogeneous Model Fitting for Multi-source Image Correspondences. Int. J. Comput. Vis..

[39] Yang W., Mei L., Ye Z., Wang Y., Hu X., Zhang Y., Yao Y. (2024). Adjacent Self-Similarity 3-D Convolution for Multimodal Image Registration. IEEE Geosci. Remote Sens. Lett..

[40] Ye Y., Yang C., Gong G., Yang P., Quan D., Li J. (2024). Robust Optical and SAR Image Matching Using Attention-Enhanced Structural Features. IEEE Trans. Geosci. Remote Sens..

[41] Yin H., Gong Y., Qiu G. Side Window Filtering. Proceedings of the 2019 IEEE/CVF Conference on Computer Vision and Pattern Recognition (CVPR).

[42] Perona P., Malik J. (1990). Scale-space and edge detection using anisotropic diffusion. IEEE Trans. Pattern Anal. Mach. Intell..

[43] Weickert J., Romeny B.M.T.H., Viergever M.A. (1998). Efficient and reliable schemes for nonlinear diffusion filtering. IEEE Trans. Image Process..

[44] Morrone M.C., Owens R.A. (1987). Feature detection from local energy. Pattern Recognit. Lett..

[45] Geusebroek, Smeulders Fragmentation in the vision of scenes. Proceedings of the Ninth IEEE International Conference on Computer Vision.

[46] Ayed I.B., Hennane N., Mitiche A. (2006). Unsupervised Variational Image Segmentation/Classification Using a Weibull Observation Model. IEEE Trans. Image Process..

[47] Wu Y., Ma W., Gong M., Su L., Jiao L. (2014). A novel point-matching algorithm based on fast sample consensus for image registration. IEEE Geosci. Remote Sens. Lett..

[48] Aguilera C.A., Sappa A.D., Toledo R. LGHD: A feature descriptor for matching across non-linear intensity variations. Proceedings of the 2015 IEEE International Conference on Image Processing (ICIP).

[49] Lan C., Lu W., Yu J., Xv Q. (2021). Deep learning algorithm for feature matching of cross modality remote sensing images. Acta Geod. Et Cartogr. Sin..

[50] Deng Y., Ma J. (2022). ReDFeat: Recoupling detection and description for multimodal feature learning. IEEE Trans. Image Process..

[51] Brown M., Süsstrunk S. Multi-spectral SIFT for scene category recognition. Proceedings of the IEEE Conference on Computer Vision and Pattern Recognition (CVPR).

